# Exploration of Simplified Intraluminal TEVAR Technique for the Treatment of Aortic Arch Disease

**DOI:** 10.21470/1678-9741-2020-0057

**Published:** 2021

**Authors:** Bailang Chen, Minxin Wei

**Affiliations:** 1 Division of Cardiovascular Surgery, The Seventh Affiliated Hospital, Sun Yat-sen University, Shenzhen, China.; 2 Department of Cardiovascular Surgery, Fuwai Hospital, Chinese Academy of Medical Sciences, Shenzhen, China.

**Keywords:** Aorta, Thoracic, Computed Tomography Angiography, Aorta, Stents

## Abstract

**Objective:**

The positional relationship between the three branches of the aortic arch was determined in normal people. This study provides data to support the customization of aortic arch stents and simplifies intraluminal treatment.

**Methods:**

From January 2019 to August 2019, 120 patients who met the inclusion criteria were examined by CT angiography. The ratio of the distance from the midpoint of the three-branch opening onto the anterior wall to the cross-sectional diameter of the aortic arch was calculated. The positional relationship among the three-branch openings was obtained and the data were analyzed statistically.

**Results:**

The three-branch openings were not in a straight line. The positional relationship among the three-branch openings was divided into four types, which were not statistically different between sex and age (*P*>0.05).

**Conclusion:**

By measuring the opening position of the three aortic branches, the positional relationship among the three branches was defined to provide a theoretical basis for the design of intraluminal stents and simplified intracavity thoracic endovascular aortic repair (TEVAR) technology.

**Table:** 

Abbreviations, acronyms & symbols	
**AA**	**= Aortic arch**
**BCT**	**= Brachiocephalic trunk**
**CTA**	**= Computed tomography angiography**
**CT**	**= Computed tomography**
**LCCA**	**= Left common carotid artery**
**LSA**	**= Left subclavian artery**
**LSD-t**	**= Least significant difference test**
**SPSS**	**= Statistical Package for the Social Sciences**
**TEVAR**	**= Thoracic endovascular aortic repair**

## INTRODUCTION

The incidence of aortic arch disease has increased annually due to an aging population and the rise of cardiovascular diseases. Dissecting aortic aneurysms are characterized by acute onset and severe illness. These patients frequently manifest several symptoms, such as sudden severe pain, shock, and organ ischemia that decrease health status and quality of life of patients^1^^).^ Standard open repair, although technically possible, is often associated with relatively high surgical trauma, postoperative mortality, and serious complications (including stroke and myocardial infarction)^2,3^. As a result, less invasive thoracic endovascular aortic repair (TEVAR) approaches have been designed to partially substitute traditional thoracotomy for the treatment of most conditions of the thoracoabdominal aorta. 

However, due to the unique anatomical structure of the aortic arch (AA), the use of endovascular approaches to repair the aortic arch has been limited by the tortuosity of the aorta and hemodynamic forces, as well as the need to maintain the perfusion of the vital arch vessels^4,5^^).^

To further broaden the surgical indications for TEVAR and simplify the surgical approach for endovascular treatment, a deeper understanding of AA morphology is needed. Most previous studies have focused on the measurement of the aortic diameter, the distance between the three branches of the AA and their angle with the AA. Although some studies have a detailed description of the aorta and AA branches, they are not relevant enough to the occurrence of aortic disease and endovascular treatment.

**Fig. 1:**
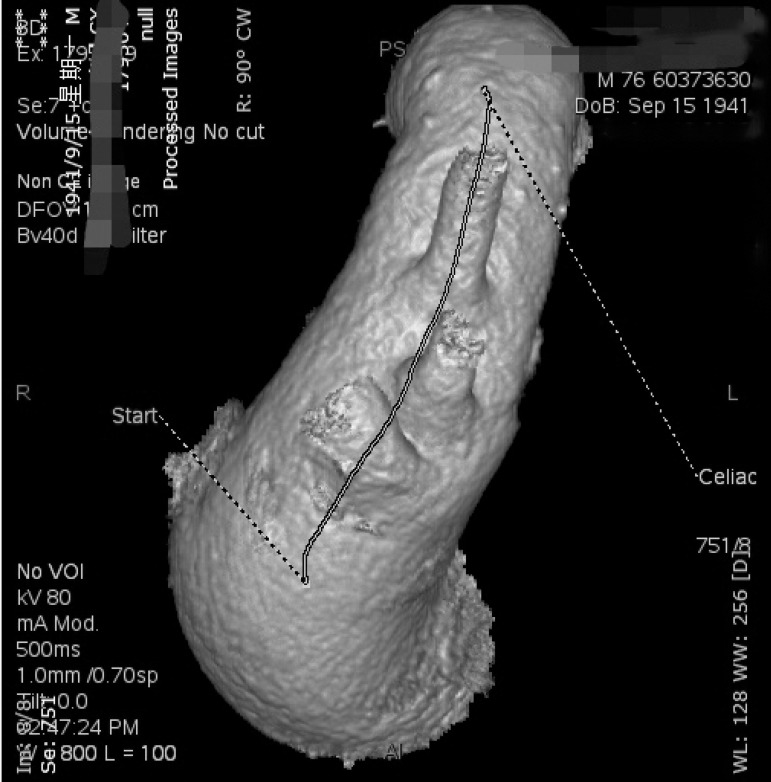
The LSA-BCT center line seems parallel to the aortic arch axis.

This study took a different perspective by using a commercially available software platform and vascular imaging workstation to define the relationships among the three arch vessels and the AA axis in patients without aneurysms or dissections (Figure 1). This provides a theoretical basis for further stent design and simplification of the treatment for aortic arch aneurysms.

## METHODS

### Patient Selection

Patients who underwent thoracic aortic computed tomography angiography (CTA) in our hospital from January to August 2019 were enrolled in this study. The CTA examination was conducted in patients with chest pain, abdominal pain, and low back pain that resulted in diagnoses of kidney stones, nerve-root type cervical spondylosis, coronary heart disease, or pulmonary embolism. 

Exclusion criteria included: 1) patients diagnosed with arterial diseases such as aortic aneurysms, aortic dissection, aortic wall hematomas, or aortic penetrating ulcers affecting the thoracic aorta and/or AA branches; 2) inadequate CT scan parameters, range, and image quality, including unsuitable window width and position, or intravascular lumen contrast agents that were poorly filled; 3) patients with severe organic lesions in the chest or mediastinum causing changes in aortic morphology; 4) patients diagnosed with connective tissue diseases, e.g. Marfan syndrome; 5) patients with aortic diseases after surgery; 6) the presence of variations in the AA branches.

After applying the exclusion criteria, 80 men and 40 women aged 16 to 89 years old, with an average of (63±14) years, were registered. 

Inspection Steps and Image Processing Methods

Before performing the CT scans, patient history of iodine allergy, renal function, and recent similar tests was collected in recent days. An allergy test was performed before the examination to observe whether the patient had an allergic reaction such as itchy skin or difficulty breathing. Scanning was carried out using a 64-row spiral CT (GE Corporation, United States), so that the scanning plane was continuous from the mandible to the groin. The scanned image was entered into an AW4.6 workstation in DICOM format. The centerline tool was utilized to straighten the AA, and the cross section of the three branches was found. The three branch positions of the AA were evaluated separately. 

### Measurement Methods

**Fig. 2:**
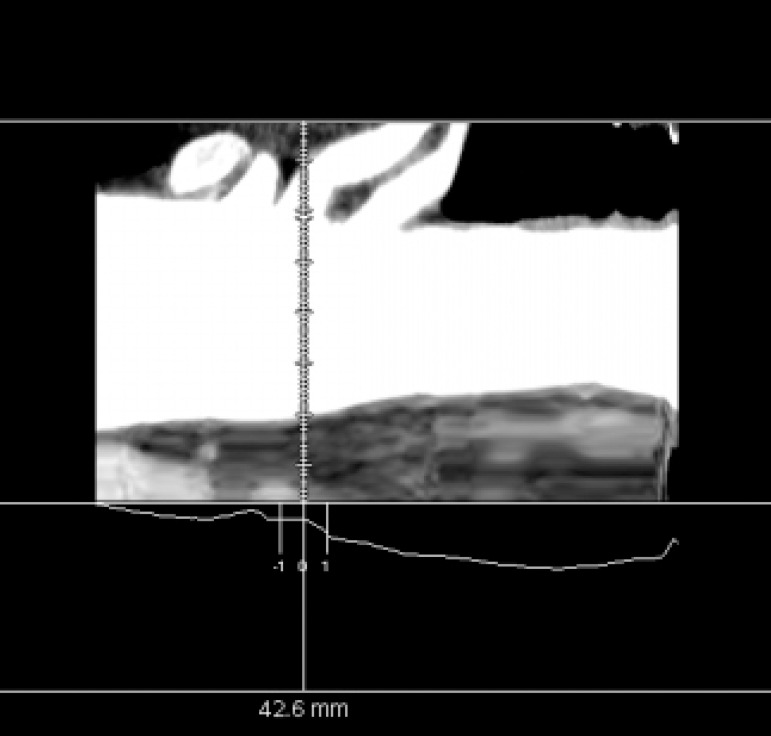
The aortic arch was straightened with the center line tool. The three branches of the aorta were observed in the image side view. The center point of the three branches was selected to define the center of a cross section.

**Fig. 3:**
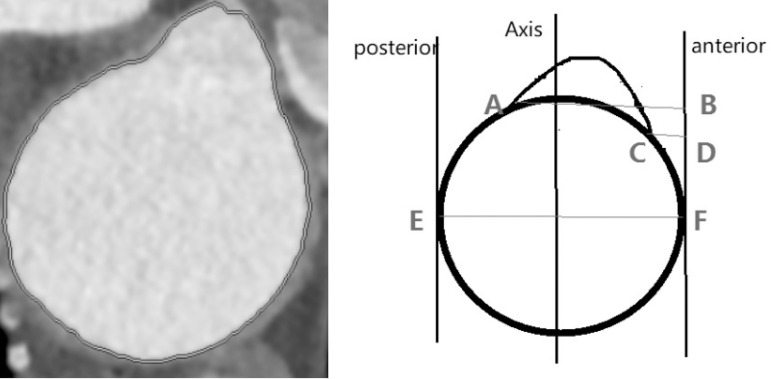
Actual three-branch cross-section diagram and schematic diagram. The intersection (A,C) of the branch opening and the aortic arch were marked on the cross-sectional image. Cross-sectional diameter was determined by EF. The line passing BD was drawn perpendicularly to EF, and the line AB, CD was parallel to EF.

The appropriate CT sequence was selected and the Vessel IQ Xpress and Vessel Analysis functions were used to obtain an image of the straightened AA. The central point of the three branches on the straightened image was identified (Figure 2). The cross section of the three branches at the central point position was determined (Figure 3). The vertical distances (AB, CD) from the anterior and posterior points A and C to the anterior wall of the aorta were measured. The diameter of the aortic arch (EF) was measured and the AB/EF and CD/EF ratios were calculated. The ratio of the distance from the center of the branch opening onto the anterior wall to the diameter of the aortic arch was defined as: ((AB/EF+CD/EF)/2). The ratios of the perpendicular distance from the center of the three branches of the left subclavian artery (LSA), left common carotid artery (LCCA), and brachiocephalic trunk (BCT) to the anterior wall and the diameter of the aortic arch in the cross section were defined as K1, K2, and K3, respectively. 

### Data Analysis

The absolute value (K1-K2, K1-K3, K2-K3) ≤0.1 was approximated as a two-branch center line parallel to the aortic arch axis, and these data were used to assess the positional relationship of the three branches of the aorta. Finally, K values and classifications were grouped by gender and age (group ≤40 years, group 41~60 years, group ≥61 years). 

### Statistical Methods

Data were analyzed using SPSS 17.0 software. The measurement data were expressed as mean±standard deviation. The t-test was used to compare sex groups. One-way analysis of variance was used to compare age groups, and pairwise post hoc multiple comparisons were performed using least significant difference tests (LSD-t). The positional relationship of the three branches of the aorta in the normal population was divided into types I, II, III and IV. The different distribution of each type in males and females or in each age group was tested by chi-square tests. A *P*<0.05 was considered statistically significant for all tests.

## RESULTS

### Three-Branch Opening Position

Descriptive data for K1, K2 and K3 are found in Table 1. The three-branch openings were not completely in a straight line. The LSA was closer to the anterior wall of the aortic arch, the LCCA was closer to the anterior wall, and the BCT was closer to the posterior wall. There were no significant differences in K1, K2 or K3 by gender (Table 2) or age (Table 3).

**Table 1:** Descriptive data for K1, K2 and K3.

Date	K1 (mm)	K2 (mm)	K3 (mm)
Average	0.487±0.060	0.458±0.051	0.530±0.071
Quartile	(0.45, 0.52)	(0.42, 0.49)	(0.48,0.58)
Median	0.48	0.46	0.52

**Table 2:** Comparison of K between gender groups.

Gender	n	K1	K2	K3
Male	80	0.484±0.065	0.455±0.054	0.530±0.074
Female	40	0.494±0.046	0.463±0.044	0.529±0.065
*P* *		0.367	0.423	0.957

* *P*>0.05

**Table 3:** Comparison of K between age groups.

Age	n	K1	K2	K3
≤40	10	0.488±0.043	0.423±0.063	0.511±0.069
41~60	31	0.476±0.049	0.465±0.058	0.517±0.078
≥61	79	0.492±0.065	0.459±0.045	0.537±0.067
*P* *		0.461	0.07	0.281

* *P*>0.05

### Classification of Three-Branch Positional Relationship

**Fig. 4:**
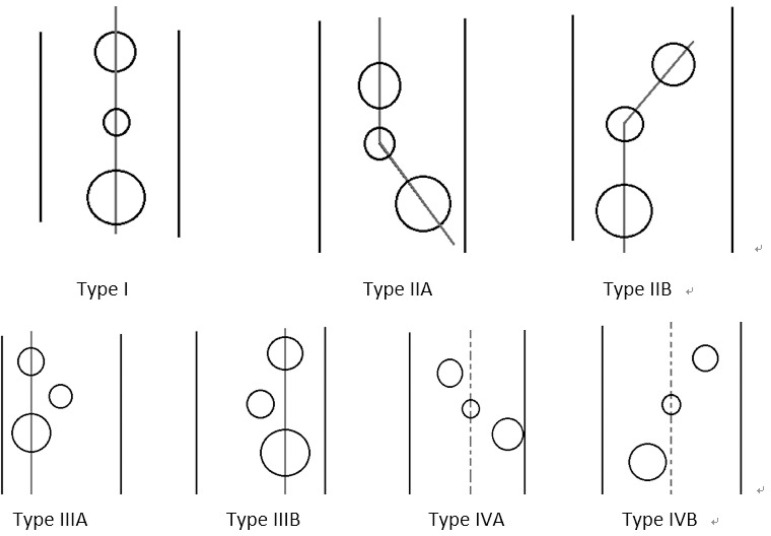
Three-branch opening classification.

Data suggest that there was a specified relationship between the three-branch openings. K1, K2, and K3 were used as the central positions of the openings of the three branches. If the absolute value (L1-L2, L2-L3, L1-L3) was less than 0.1, the center line of the two branches was defined as parallel to the axis of the aortic arch. The morphological differences were divided into four types: type I (71 cases, 59.2%): the three-branch straight center line was approximately parallel to the aortic arch axis; type II (29 cases, 31.7%): the LSA-LCCA center line was parallel to the aortic arch axis and was defined as type IIA (the BCT-LCCA center line was parallel to the aortic arch axis and was defined as type IIB); type III (10 cases, 8.3%): the LSA-LCCA center line was parallel to the aortic arch axis and was defined as type III (if the LCCA was closer to the anterior wall of the aortic arch, it was defined as type IIIA, and if it was closer to the posterior wall, it was defined as type IIIB); type IV (one case, 0.8%): there was no parallel relationship with the aortic arch axis among the three-branch openings (Figure 4). There were no statistical differences among the four types with gender or age (Tables 4 and 5).

**Table 4:** Comparison of types between gender groups.

Gender	I	II	III	IV	Total
Male	47(47.3)	25(25.3)	7(6.7)	1(0.7)	80(80.0)
Female	24(23.7)	13(12.7)	3(3.3)	0(0.3)	40(40.0)
Total	71(71.0)	38(38.0)	10(10.0)	1(1.0)	120(120.0)

*P*=1.000>0.05. There was no statistically significant difference in the positional relationship of the three branches of the aortic arch in the gender group, as shown in *Table 4*.

**Table 5:** Comparison of types between age groups.

Age	I	II	III	IV	Total
≤40	5(5.9)	2(3.2)	3(0.8)	0(0.1)	10(10.0)
41-60	17(17.8)	9(9.5)	4(2.5)	0(0.3)	30(30.0)
≥61	49(47.3)	27(25.3)	3(6.7)	1(0.7)	80(80.0)
Total	71(71.0)	38(38.0)	10(10.0)	1(1.0)	120(120.0)

*P*=0.116>0.05. There were no significant differences in the classification of the position of the three branches of the aortic arch in the three age groups ≤40 years old, 41-60 years old, and ≥61 years old, as shown in *Table 5*.

### Stent Design Model

At present, the standard length of the front end of the stent branch is 5, 10, 15, or 20 mm. In theory, the greater the distance between the LSA and the LCCA, the greater the length of the front-end stent will have a longer anchoring zone, reducing leakage and displacement of the stent. The front end of the stent branch has different specifications. In addition, different patients have different aortic diameters, so there are more than 10 types of single-branched stents. Therefore, the manufacture of such a stent is more cumbersome. 

Data showed that the central connection between the LSA and the LCCA was approximately parallel to the axis of the aortic arch, accounting for more than 80% of the cases. Therefore, we designed a groove in the front end of a single branch bracket. In this way, different length specifications at the head end of the stent due to the distance uncertainty between the LSA and the LCCA are unnecessary. Significantly reduced size of the single-branch bracket facilitates production. 

**Fig. 5:**
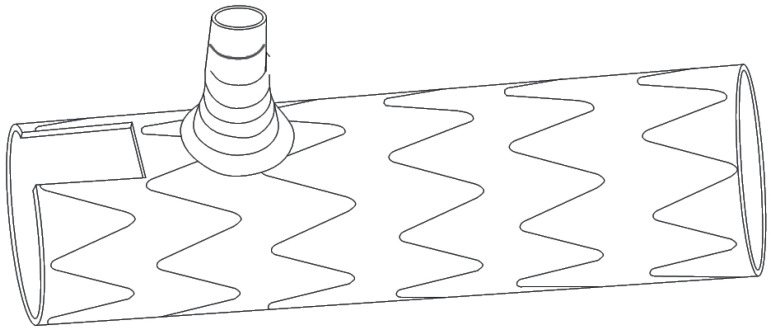
Single-branch bracket model slotted at the front end of the bracket.

**Fig. 6:**
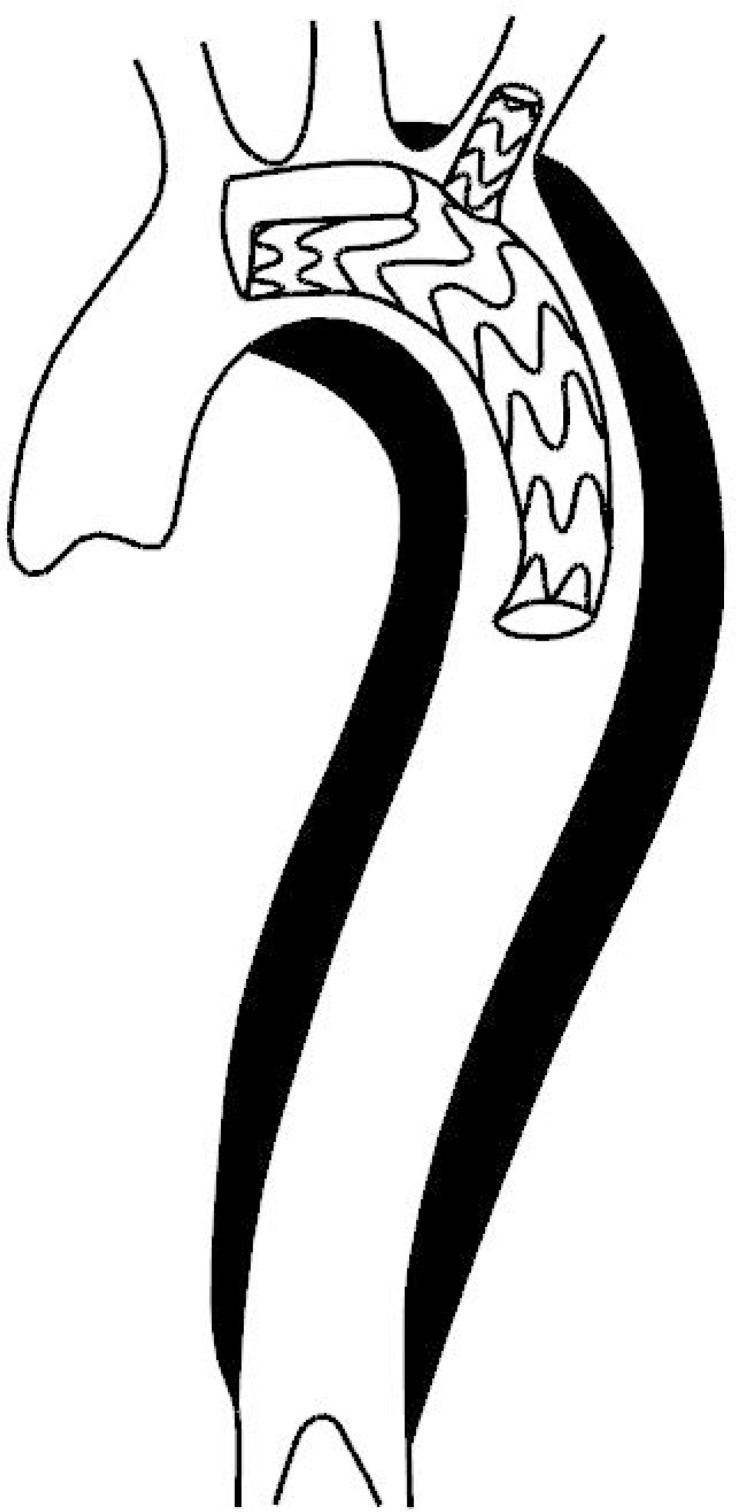
The stent excluded the hematoma and ensured the patency of the branch vessels.

As shown in Figure 5, the groove is slotted at the head end of the single branch, and the center line of the groove and branch is parallel to the axis of the stent. The bottom edge of the groove is as long as the branch diameter. This model does not need to consider the distance between the LSA and the LCCA. The model avoids LCCA closure and greatly simplifies the design of single-branch brackets. It is convenient to produce and stock (Figures 5 and 6).

## DISCUSSION

In the past, the positional relationship of the three branches of the aortic arch (AA) was regarded complex and varied from person to person. Such characteristics limit the current application of aortic arch stents. We determined the regularity of the three branches by measuring their positional relationship and divided them into four types. These data can be used to improve the existing stent model, simplifying the production specifications of the stents.

Although some studies have revealed the anatomy of the AA^6,7^^),^ there are few morphological studies associated with endovascular treatment^8^. In previous studies, the positional relationship between the three branches of the aorta and the AA was concentrated mainly on the distance between the three branches and the angle between the three branches and the axis of the AA. Finlay et al.^9)^ showed that the distance from the aortic sinus to the brachiocephalic trunk (BCT), the left common carotid artery (LCCA), and the left subclavian artery (LSA) were 69.9±11.8 mm, 8 1.7±13.8 mm, 9 6.6±15.8 mm, respectively. The distances from BCT to LCCA and from LCCA to the LSA were 5.1±1.5 mm and 10.9±4.4 mm, respectively. Yu et al.^10^ showed that the distance between BCT and LCCA was 4.39±2.49 mm, and the distance between LCCA and LSA was 6.43±3.98. Shin et al.^11^ studied 25 cases at autopsy and found that the angles between BCT, LCCA, LSA and the AA were 65.3º, 46.9º, 63.8º, respectively.

Measurement of relevant data provides parameters for the design of the stents. The data vary due to individual differences among people. Previous studies have not been able to customize AA stents because of the variability of the three branches of AA. There may be certain rules between the three-branch openings, which are important to better understand the morphology of the AA branches. In this study, we determined the positional relationship between the three-branch openings of the AA from a different perspective. By measuring the opening position of the three branches, we can better understand the positional relationship among the three branches to provide a theoretical basis for the design of intraluminal stents and to simplify the intracavitary TEVAR technology.

Multidetector CT has become the most common method for evaluating thoracic vasculature^12)^ and the main diagnostic method for assessment of thoracic aortic abnormalities^13^^).^ It is increasingly used to assess the morphology of AA. Due to the natural curved structure of the aortic arch, it is difficult to measure the position of the three branches of the AA. In this study, we utilized the centerline tool to more accurately measure the position of the AA branches on the cross-sectional image.

There seems to be a specific and well-defined relationship among the three branches, which does not differ by sex or age. The aortic three-branch positional relationship can be divided into four types. The different morphological types have nothing to do with age and gender but may be determined in part genetically.

The castor device employed an easy-to-use unibody design, including a main body and a branch graft to avoid type III endoleaks. Clinicians have been successful in a small number of cases using self-made or customized branch stents and achieved satisfactory clinical results^14,15^^).^ Due to the difference in distance between the LSA and the LCCA, previous single-branch stent models are often designed to different specifications to avoid LCCA closure. Compared to the traditional single-branch stent, the new stent model does not need to consider the distance between the LCCA and the LSA, which simplifies the stent design and is more convenient for clinical application. The parallel relationship between the aortic branches we found provides the theoretical basis for simplifying the stent design. Such production of standardized grafts will reduce manufacturing costs and shorten the present delay in therapy that exists with customized production. 

### Limitations

Our study is based on a normal three-branch population, and we have not conducted a thorough study of the population of variant aortic branches. The population we selected was mainly in the hospital. Patients with a CTA exam for various reasons have a certain bias. In the future, we will need to recruit non-hospital populations for measurement and observation and collect multicenter and multiregional data so that the results can be more refined to reflect the general population.

## CONCLUSIONS

The development of standardized off-the-shelf aortic arch endografts will reduce production costs and treatment delays that currently place patients at an additional risk of adverse sequelae. The present study describes a stent model based on the results of a morphological study. Based on these findings, a prototype of an off-the-shelf endograft is suggested that can now be evaluated, refined, and validated by future studies.

### Authors’ Note

This investigation was reviewed and approved by the Seventh Affiliated Hospital, Sun Yat-sen University institutional review board, following the principles outlined in the Declaration of Helsinki. A waiver for informed consent was granted due to the study’s minimal risk and retrospective methodology.

**Table:** 

Authors' roles & responsibilities	
BC	Substantial contributions to the conception or design of the work; or the acquisition, analysis, or interpretation of data for the work; drafting the work or revising it critically for important intellectual content; final approval of the version to be published
MW	Substantial contributions to the conception or design of the work; or the acquisition, analysis, or interpretation of data for the work; drafting the work or revising it critically for important intellectual content; final approval of the version to be published
